# Validity and Reliability of a Self-Reported Measure of Antihypertensive Medication Adherence in Uganda

**DOI:** 10.1371/journal.pone.0158499

**Published:** 2016-07-01

**Authors:** Samson Okello, Benson Nasasira, Anthony Ndichu Wa Muiru, Anthony Muyingo

**Affiliations:** 1 Department of Internal Medicine, Mbarara University of Science and Technology, Mbarara, Uganda; 2 Department of Epidemiology, Harvard T.H. Chan School of Public Health, Boston, Massachusetts, United States of America; 3 Department of Medicine, Massachusetts General Hospital, Boston, Massachusetts, United States of America; University of Perugia, ITALY

## Abstract

**Background:**

The Morisky Medication Adherence scale (MMAS-8) is a widely used self-reported measure of adherence to antihypertensive medications that has not been validated in hypertensive patients in sub-Saharan Africa.

**Methods:**

We carried out a cross-sectional study to examine psychometric properties of a translated MMAS-8 (MMAS-U) in a tertiary care hypertension clinic in Uganda. We administered the MMAS-U to consecutively selected hypertensive adults and used principal factor analysis and Cronbach’s alpha to determine its validity and internal consistency respectively. Then we randomly selected one-sixth of participants for a 2-week test-retest telephone interview. Lastly, we used ordinal logistic regression modeling to explore factors associated with levels of medication adherence.

**Results:**

Of the 329 participants, 228 (69%) were females, median age of 55 years [Interquartile range (IQR) (46–66)], and median duration of hypertension of 4 years [IQR (2–8)]. The adherence levels were low (MMAS-U score ≤ 5) in 85%, moderate (MMAS-U score 6–7) in 12% and high (MMAS-U score ≥8) in 3%. The factor analysis of construct validity was good (overall Kaiser’s measure of sampling adequacy for residuals of 0.72) and identified unidimensionality of MMAS-U. The internal consistency of MMAS-U was moderate (Cronbach α = 0.65), and test-retest reliability was low (weighted kappa = 0.36; 95% CI -0.01, 0.73). Age of 40 years or greater was associated with low medication adherence (p = 0.02) whereas a family member buying medication for participants (p = 0.02) and purchasing medication from a private clinic (p = 0.02) were associated with high adherence.

**Conclusion:**

The Ugandan version of the MMAS-8 (MMAS-U) is a valid and reliable measure of adherence to antihypertensive medication among Ugandan outpatients receiving care at a public tertiary facility. Though the limited supply of medication affected adherence, this easy to use tool can be adapted to assess medication adherence among adults with hypertension in Uganda.

## Introduction

Globally, high blood pressure is the leading risk factor for morbidity and accounts for 7% of global disability-adjusted life years (DALYs) and nearly 10 million deaths per year [1]. Despite global declines in blood pressure, the blood pressures of adults in sub-Saharan Africa (SSA) continue to rise [2, 3], and the age-adjusted prevalence of hypertension in SSA is estimated to be the highest of any region in the world [4]. In fact, Ugandan community-based prevalence studies have shown a prevalence of hypertension ranging from 20–27% [5–7].

Adherence to antihypertensive medications is necessary in order to achieve blood pressure control, and improve outcomes [8, 9]. However, Uganda faces unique challenges in achieving blood pressure control partly because the health care system is ill equipped to address the rising burden of non-communicable diseases [10]. As seen in other SSA countries, there are vast socioeconomic barriers, inequalities in access to treatment, suboptimal staffing in health-care facilities, limited supply of medication, and limited capacity to conduct clinical investigations [11–13]. In order for health care providers to promote medication adherence, an easy to use, reliable and valid measure of medication adherence is needed.

The 8-item Morisky Medication Adherence (MMAS-8) scale is a low cost, simple and self-reported tool for assessment of adherence to chronic medications specifically designed to facilitate identification of barriers to antihypertensive medication adherence in real-time, which is critical in clinical practice [14, 15]. Though the MMAS-8 has been shown to have a 93% sensitivity and 53% specificity among very low income minority hypertensive patients seeking routine care in a clinic setting in the United States [15], further refinement and consistent demonstration of validity and reliability in resource limited settings are needed before adoption. In this study, we sought to assess validity, internal consistency and test-retest reliability of the MMAS-8 for measurement of adherence to antihypertensive medication and to explore factors associated with low adherence in a large public funded hypertension care facility in Uganda.

## Methods

### Design and setting

This cross sectional study was conducted at the Mbarara Regional Referral Hospital (MRRH) hypertension clinic. This outpatient clinic is publicly funded, and provides clinical care for over 3000 patients from within and as far as 80 KM away who thus incur the cost of transportation in order to obtain care at MRRH. The MRRH hypertension clinic serves on average 120 patients every week with follow-up visits for hypertensive patients ranging from every three weeks to four months depending on whether the clinic blood pressure (BP) is controlled or not according to the eighth report of the joint national committee on prevention, detection, evaluation, and treatment of high blood pressure (JNC 8) [16]. Separately, the MRRH central pharmacy provides free medications including Bendroflumethiazide, Nifedipine, Amlodipine, Captopril, and Lisinopril. However, these drugs are often in short supply therefore patients are supplied with medications for a maximum two weeks prescription when in stock. Patients who can afford often purchase prescribed medications out of pocket in privately- owned pharmacies or clinics.

### Participant recruitment

We consecutively screened patients attending the MRRH hypertension clinic to enroll participants who had been enrolled in the clinic at least 6 months prior to this study, and filled a prescription of antihypertensive therapy at least once within 2 weeks prior to this study. The 6-month period was chosen to identify participants with sufficient exposure time to antihypertensive therapy whereas the requirement for a refill within 2 weeks prior to study selection was used to ensure recent availability of medication since the MMAS-8 is designed to assess recent medication-taking behavior [17].

Participant recruitment occurred between January and May 2015. Eligible participants were consecutively selected from the clinic attendance register on each clinic day after general counseling sessions. A trained nurse, who had just been recruited for this study and unknown to majority of patients, sought consent from consecutive patients after description of the study. The new nurse was employed so as to reduce the social desirability bias that would occur if we used a clinic nurse known to patients.

The same nurse re-surveyed, by telephone interviews, a randomly selected subset of participants within 2 weeks for test-retest reliability assessment of MMAS-U with an a priori goal of having a-sixth of participants complete the second survey. The participants for re-interview were selected by generating a random sample from all enrolled participants using SAS statistical software. Telephone interviews were conducted in the order the initial survey was administered.

### Data collection

The study nurse collected information on socio-demographic characteristics, highest education level attained, occupation, marital status, average monthly income, time in months/years since diagnosis of hypertension, travel time to reach clinic, distance travelled to reach clinic, mode of transportation to clinic, cost of transportation to clinic, and history of comorbid conditions. Self-reported medication adherence was assessed using the translated MMAS-U scale. Scoring for the self-reported instruments was performed according to the developer’s guidelines [15].

### Blood pressure measurement

At each clinic visits, all patients’ anthropometric and blood pressure measurements are performed by the clinic nurses before their consultation with doctors. Using standardized forms, a second study nurse (blinded to participant MMAS-U scale adherence category) extracted systolic and diastolic blood pressure measurements from the medical records on that day and the clinic visit immediately before the survey. Blood pressure were averaged for the various visits when more than one measurement was found. Then, the average BP across these visits was calculated and used to defined blood pressure control as per JNC 8 guidelines [16].

### The 8-item Morisky medication adherence scale (MMAS-8)

The MMAS-8 is self-report questionnaire with 8 questions (items) whose wording of the questions/items are formulated to avoid answering “yes” to questions regardless of their content. Items 1 through 7 have response choices “yes” or “no” whereas item 8 has a 5-point Likert response choices. Each ‘‘no” response is rated as ‘‘1” and each ‘‘yes” is rated as ‘‘0” except for item 5, in which each response ‘‘yes” is rated as ‘‘1” and each ‘‘no” is rated as ‘‘0”. For item 8, if a patient chooses response ‘‘0”, the score is ‘‘1” and if they choose response ‘‘4”, the score is ‘‘0”. Responses ‘‘1, 2, 3” are respectively rated as ‘‘0.25, 0.75, 0.75”. Total MMAS-8 scores can range from 0 to 8 and have been categorized into three levels of adherence: high adherence (score = 8), medium adherence (score of 6 to < 8), and low adherence (score< 6) [18].

### Translation of the Morisky Medication Adherence scale

Two translators independently translated the English MMAS-8 to Runyankore/Rukiga; the dominant and widely spoken indigenous language in southwestern Uganda. Another bilingual translator, who was not involved in developing the initial version, performed reverse translation from Runyankore/Rukiga to English. The original and the back-translated English versions were compared and inconsistencies were resolved by consensus. A pilot test was performed in 10 subjects to ensure understanding of the wording of the Runyankore/Rukiga version and no inconsistencies were revealed. The subjects who participated in this pilot face-validity phase were not included in the study.

We set, a priori, a minimum 200 patients based on the ratio (sample size: number of items) of 20:1 being adequate to produce correct factorial structure [19] with a tight 95% confidence bound for a Cronbach alpha coefficient of 0.80 [20] for an 8-item MMAS questionnaire.

All patients provided individual-level consent and this study was approved by the Institutional Review Committee at Mbarara University of Science and Technology, Uganda.

### Statistical analysis

We summarized continuous variables by means and standard deviation, or medians and interquartile range, and categorical variables summarized by proportions. Age, distance away from clinic and average monthly cost on medication were modeled and compared independently using Akaike (AIC)’s criteria to determine the form of the variables that yields the best model fit (lowest AIC).

We assessed the construct validity of the questionnaire using principal component analysis with varimax rotation while the number of components retained in the component analysis was examined using principal factor analysis and the internal consistency of the MMAS-U questionnaire using Cronbach alpha coefficient. We used kappa agreement to assess test-retest reliability at a 2-week interval.

We then performed an exploratory multivariate ordinal logistic regression analysis to evaluate association of demographic and clinical variables with each ordinal change in adherence level (low, medium, and high). The adjusted odds ratio (AOR) corresponds with the odds of adherence in the next level according to the MMAS-U. We set out a priori to include male gender and distance away from clinic in all models basing on prior knowledge of this being the common factors associated with low adherence. A p-value threshold of 0.05 was used to assess for statistically significant associations. All analyses were preformed using SAS statistical software (SAS Institute Inc, Cary, NC; release 9.4).

## Results

Of the 890 eligible patients who visited the clinic during the study period, 331 (37.2%) were consecutively selected to participate in this study; of these 2 (0.6%) withdrew consent before completing the survey because of insufficient time for participation. Of the remaining 329, there were 228 (69%) females with a median age of 55 years [Interquartile range (IQR) (46–66)]. Socio-demographic and lifestyle characteristics of the participants are presented according to the MMAS-U adherence levels in Table 1.

**Table 1:** Baseline characteristics of hypertensive patients.

Characteristic	Low adherence, 280 (85%)	Moderate adherence, 40 (12%)	High adherence, 9 (3%)
Female	192 (68.57)	28 (70)	8 (88.89)
Age			
< 40	38 (13.57)	9 (22.50)	1 (11.11)
40–50	66 (23.57)	7 (17.50)	2 (22.22)
51–60	77 (27.50)	6 (15.00)	2 (22.22)
> 60	99 (35.36)	18 (45.00)	4 (44.44)
Education			
No education	110 (39.29)	17 (42.50)	1 (11.11)
Primary	112 (40.00)	17 (42.50)	6 (66.67)
Secondary or higher	58 (20.71)	6 (15.00)	2 (22.22)
Marital status			
Single	10 (3.57)	2 (5.00)	-
Married	177 (63.21)	26 (65.00)	3 (33.33)
Divorced/separated	42 (15.00)	4 (10.00)	1 (11.11)
Widowed/widower	51 (18.21)	8 (20.00)	5 (55.56)
Time to clinic (hours)	1 (0.5–1)	1 (0.5–1.75)	0.5 (0.5–1)
Distance to clinic (km)	2 (1–18)	5.5 (2–21)	15 (5–30)
Mode of transport to clinic			
Walking	12 (4.29)	3 (7.50)	-
Car taxi	173 (61.78)	23 (57.50)	4 (44.44)
Motor cycle taxi	95 (33.93)	14 (35.00)	5 (55.56)
Self reported monthly Income quintiles			
Poorest, n (%)	69 (24.64)	15 (37.50)	3 (33.33)
Poor, n (%)	58 (20.71)	12 (30.00)	2 (22.22)
Average, n (%)	30 (10.71)	4 (10.00)	3 (33.33)
Rich, n (%)	53 (18.93)	5 (12.50)	1 (11.11)
Declined to respond	70 (25.00)	4 (10.00)	-

Overall, 85, 12, and 3% of study participants had low adherence (score <6), medium adherence (scores 6 to 7), and high adherence (score > 8 or equal) on the MMAS-U tool respectively. Participants in lower adherence scores were likely to have a comorbid condition, reported being hospitalized within 6 months prior to survey, and receiving antihypertensive medication only in MRRH central pharmacy (Table 2).

**Table 2:** Clinical characteristics according to medication adherence scores.

Characteristic	Low adherence 280 (85%)	Medium adherence 40 (12%)	High adherence 9 (3%)
Comorbidity	161 (57.50)	17 (42.50)	4 (44.44)
Hospitalized within last 6months	133 (47.50)	15 (37.50)	1 (11.11)
Controlled (SBP <140 and or DBP < 80 mmHg)	174 (62.14)	30 (75.00)	8 (88.89)
Number of medication classes			
1	37 (13.21)	6 (15.00)	2 (22.22)
2–3	122 (43.57)	17 (42.50)	7 (77.77)
> 4	121 (43.22)	17 (42.50)	-
Where medication was received			
Government facility	207 (73.93)	22 (55.00)	6 (66.67)
Private pharmacy	66 (23.57)	14 (35.00)	3 (33.33)
Private clinic	3 (1.07)	2 (5.00)	-
Other	7 (2.5)	2 (5.00)	-
Duration of treatment (years)			
< 1	38 (13.57)	7 (17.50)	2 (22.22)
1–2	35 (12.50)	4 (10.00)	-
2–5	109 (38.93)	11 (27.50)	3 (33.33)
≥ 5	98 (35.00)	18 (45.00)	4 (44.44)

### Construct validity of the MMAS-U

Confirmatory factor analysis indicated that the 8-items of the MMAS-U loaded on two factors, but, as can be observed, results tended to a one-factor solution: using an item selection criterion of ≥ 0.40 loading coefficients, only one item did not fall within the one-factor solution. It was Item 7, which dealt with an emotional aspect of adherence. The item loadings ranged from 0.06 to 0.81 (Table 3). The overall Kaiser’s measure of sampling adequacy for residuals was 0.72, overall RMS off diagonal partials was 0.048, and the total final community estimates was 2.53 indicating good model fit.

**Table 3:** Exploratory Factor Analysis of the Ugandan Morisky medication adherence scale in hypertensive patients.

MMAS-U Item	Yes response	No response	Factor 1	Factor 2
Item # 1	239 (72.42)	91 (27.58)	**0.694**	-0.130
Item # 2	251 (76.29)	78 (23.71)	**0.781**	0.251
Item # 3	272 (82.67)	57 (17.33)	**0.676**	0.289
Item # 4	270 (82.07)	59 (17.93)	**0.582**	-0.113
Item # 5	30 (9.12)	299 (90.88)	-0.400	0.247
Item # 6	258 (78.42)	71 (21.58)	**0.468**	0.067
Item # 7	141 (42.86)	188 (57.14)	0.092	**0.811**
Item # 8	Never/rarely	201(61.09)	**0.633**	-0.383
	Once in a while	81 (24.62)		
	Sometimes	31 (9.42)		
	Usually	15 (4.56)		
	All the time	1 (0.30)		

### Internal consistency of the translated MMAS-8 (MMAS-U)

Cronbach’s alpha for the MMAS-U was 0.65, and the deletion of any item did not reduce the Cronbach’s alpha substantially. The item-total correlations ranged from 0.90 (item 5) to 0.17 (item 3). Standardized Cronbach alpha coefficient was slightly higher 0.66 when item 5 was not used for computation. There was no significant association between adherence category and blood pressure control (p = 0.06) likely due to the small sample size thus this study was not powered for this comparison.

### Test-retest reliability and concordance of individual items on MMAS-U

A total of 52 participants completed the second MMAS-U administration within 14 days of the first assessment. Using the recommended cut-offs, 2% (1 of 52), 2% (1 of 52), and 98% (50 of 52) of patients were in the high, medium, and low adherence groups, respectively. The mean (SD) of the MMAS-U score in the retest was 2.94 (0.31). The test-retest reliability was low (weighted kappa = 0.36; 95% CI -0.01, 0.73; p<0.001). No participant had high adherence upon the first questionnaire administration and low adherence for the second administration. Ten percent of the participants had medium adherence at first questionnaire but low adherence at the second questionnaire administration.

### Factors associated with high adherence to antihypertensive medication

In an exploratory multivariable ordinal logistic regression analysis, there was a statistically significant increase in risk of low adherence with age greater than 40 years (p = 0.02) but not > 60 (p = 0.19) while purchasing medication from a private clinic (p = 0.022) or a family member buying medication for participant (p = 0.018) were associated with high medication adherence (Table 4).

**Table 4:** Multivariate ordinal logistic model of factors associated with low adherence to antihypertensive medication.

Characteristic	AOR 95%CI	p-value
Male	1.21 (0.41–3.59)	0.728
Age (years)		
< 40	Ref	
40–50	6.13 (1.32–28.44)	0.021
51–60	5.31 (1.25–22.47)	0.023
>60	2.32 (0.66–8.16)	0.189
Education		
None	Ref	
Primary	9.44 (0.33–12.74)	0.916
Secondary	7.32 (0.69–77.68)	0.099
Vocational	1.26 (0.20–7.82)	0.804
University	0.13 (0.01–1.86)	0.134
Marital status		
Single	Ref	
Married	1.78 (0.26–12.40)	0.558
Separated/Divorced	3.84 (0.29–49.87)	0.304
Widowed	0.64 (0.07–5.58)	0.685
Distance away from Clinic (Km)		
< 5km	Ref	
5–10 km	1.09 (0.22–5.52)	0.917
10–20km	0.51 (0.13–2.02)	0.339
> 20km	0.83 (0.24–2.85)	0.771
Average monthly cost on medication (thousand Uganda shillings)		
< 20	Ref	
20–50	1.01 (0.27–3.77)	0.987
50–100	3.02 (0.77–11.80)	0.111
100–500	0.54 (0.06–4.91)	0.587
Source of medication		
Public hospital pharmacy	Ref	
Private pharmacy	0.58 (0.19–1.79)	0.348
Private clinic	0.06 (0.01–0.67)	0.022
Family member bought	0.03 (0.001–0.54)	0.018

Participants reported reasons for non-adherence to medication as expensive medication in private pharmacies and clinics (48.6%), long distance to clinic (25%), service related delays at MRRH (7%), high pill burden (6.1%), running out of medication at home (5%), medication side effects (5%), forgetfulness (4%), receiving limited instructions from their health care providers and short consultation time in <1%.

## Discussion

In the present study, we show that the Ugandan version of the MMAS-8 is a valid and reliable measure of antihypertensive medication adherence among Ugandan outpatients receiving care in a public tertiary facility. The validity (the overall overall Kaiser’s measure of sampling adequacy for residuals of 0.72) in this study was consistent with those of previous studies [15, 21–23]. In contrast, factor analysis for construct validity revealed unidimensional factor loading similar to the original English 8-item MMAS [15]. Further scrutiny shows that factor loadings on items about forgetting were highest (items 1, 2, 3, 4, and 8). This is supported by the fact that forgetfulness was a common reason for non-adherence to medication in this sample. Item 6 (‘*When you feel like your blood pressure is under control*, *do you sometimes stop taking your medicine*?’) loaded least, reinforcing the fact that people tended to stop taking medication based on how they felt [24] thus patient counseling should in addition inform patients to continue taking medication and seek medical advise despite how the patients feel instead of stopping by themselves. This approach will minimize intentional circumstances that affect medication adherence. Conversely, Item 5 *(“Did you take your high blood pressure medicine yesterday*?*”)*, intended to capture unintentional factor, loaded <0.4 on both factors might have been decided based on the expected benefits of medication [25] while item 7 *(‘Taking medication everyday is a real inconvenience for some people*. *Do you ever feel hassled about sticking to your high blood pressure treatment plan*?*”*) an intentional factor, loading onto the second factor could be interpreted as a consequence of cognitive function deterioration, which interferes with the ability to remember to take medication.

We posit that multiple factors including limited supply of antihypertensive medication might have affected adherence and poor memory. In fact, majority of participants (85%) had low adherence, about 10% participants had medium adherence at first questionnaire but low adherence at the second questionnaire administration. This is expected since most participants received medication solely from the hospital central pharmacy where medications are rationed and stock outs are rampant. However, the fact that no participant had high adherence upon the first questionnaire administration and low adherence for the second administration demonstrates the robustness nature of the MMAS-8 score as a valid instrument to measure adherence even in settings with limited medication supply [26].

We observed a moderate internal consistency of the MMAS-U (Cronbach’s alpha score of 0.66), which implies that this tool can detect various levels of antihypertensive medication adherence among patients receiving care in a public tertiary care center in southwestern Uganda. This is comparable to other non-English language versions of the MMAS-8 in hypertensive patients elsewhere [27–30] and diabetes mellitus patients in Thai (0.61)[31], Malay (0.67)[23]. This is not surprising because the Cronbach’s alpha is based on the correlation between items and the number of items in a scale [32], which remained the same as in other studies.

We however observed a lower reliability score (Kappa = 0.36) when compared to the original MMAS-8 (0.83)[15]. These differences exemplify the fact that reliability of scales like the MMAS-8 medication adherence scale depend on health care practices, culture and education level of participants. We posit that we observed a lower test-retest reliability score because of the common practice of rationing antihypertensive medications at MRRH central pharmacy. Participants may have not had enough supply of medications. In fact, < 40% of the retest sample reported having antihypertensive medication at time of second administration of questionnaire.

Our exploratory analysis of factors associated with poor adherence by multivariable ordinal logistic modeling indicated age > 40 years, provision of medication by family and purchasing medication from a private clinic were associated with higher adherence. The finding of increasing age as associated with higher adherence is consistent with prior studies that report similar results in hypertensive patients in other settings [33, 34]. However, the result of family providing medication maybe explained by the fact that family support is important for adherence to medication [35]. Unlike most public facilities, private clinics endeavor to provide health education, which is known to encourage adherence to medication [36], it is therefore not surprising that purchasing medication from a private clinic was associated with high adherence. It is also likely that patients who can afford medications in private clinics often have a more consistent supply of medication compared to those who depend entirely on free medication in the public facility and thus adherence would be higher in the former.

Our data should be interpreted in the context of the study design. These results could be biased by social desirability and outcome misclassification. We attempted to minimize these by having a recently recruited nurse unknown to the participants administering the questionnaires and using standard cut-off values of MMAS-8 score to define the various levels of adherence. Also, this study was conducted in among low–income patients treated for hypertension seeking routine care in a public facility setting and may not be representative of patients from other socioeconomic backgrounds within southwestern Uganda.

However, our sample is representative and characteristic of the majority of the rural population in most sub-Saharan Africa receiving care in public funded health facilities. Future studies should consider including urban populations and private facilities to elucidate factors that contribute to medication adherence or the influence of multiple factors such access to health care services, lifestyle and environment on blood pressure control.

## Conclusion

Low adherence to antihypertensive medications is rampant, perhaps due to the limited supply of medication, in a public funded health care facility in Uganda. There is need to improve supply of antihypertensive medication to improve adherence and control of blood pressure which may reduce the long term costs, morbidity and mortality related to hypertension and its’ complications.

The Ugandan version of the MMAS-8 is a valid and reliable measure of antihypertensive medication adherence among Ugandan outpatients receiving care in a public tertiary facility. This easy to use scale can be adapted for routine assessment of medication adherence among adults with hypertension in resource-limited settings like Uganda.
