# Correction: Validity and Reliability of a Self-Reported Measure of Antihypertensive Medication Adherence in Uganda

**DOI:** 10.1371/journal.pone.0187620

**Published:** 2017-10-31

**Authors:** The PLOS ONE Staff

## Notice of Republication

The S1 Table was included in the article without full permission. The table was removed from the HTML and PDF versions of this article on May 30, 2017. Please download this article again to view the correct version. Readers can access the MMAS-8 scale through the original publication [2].
